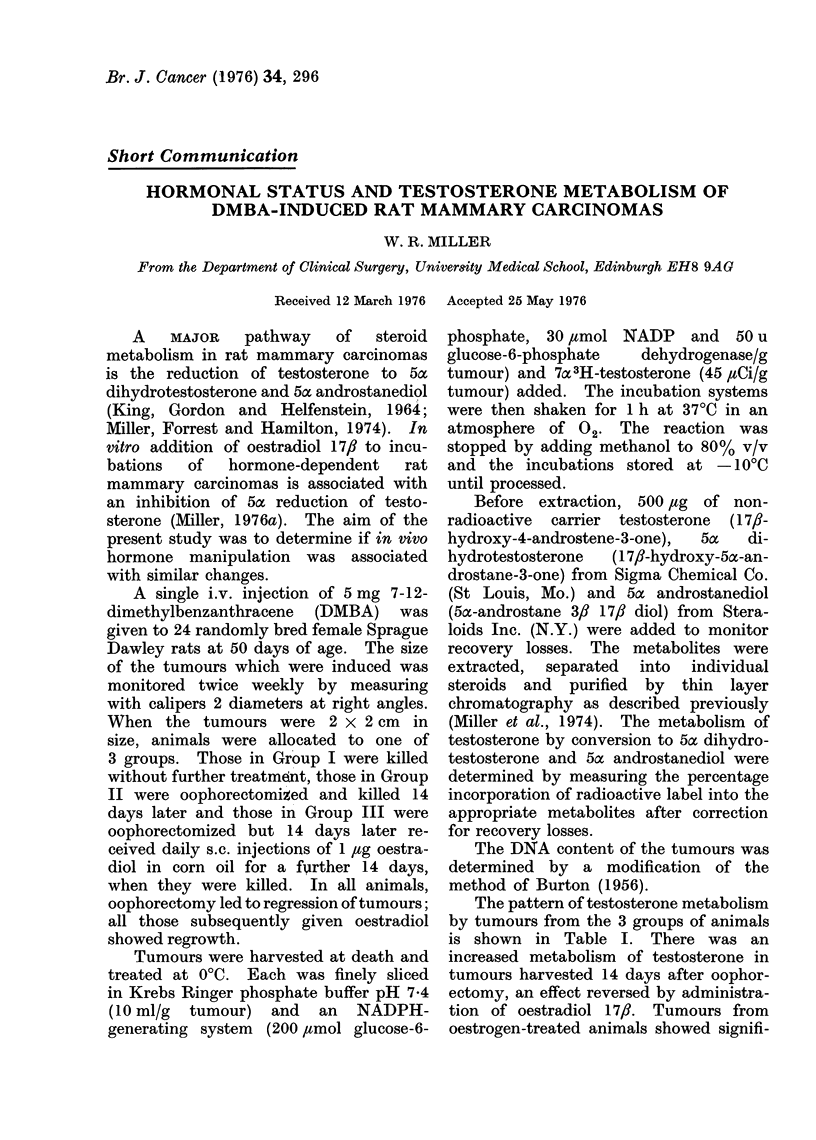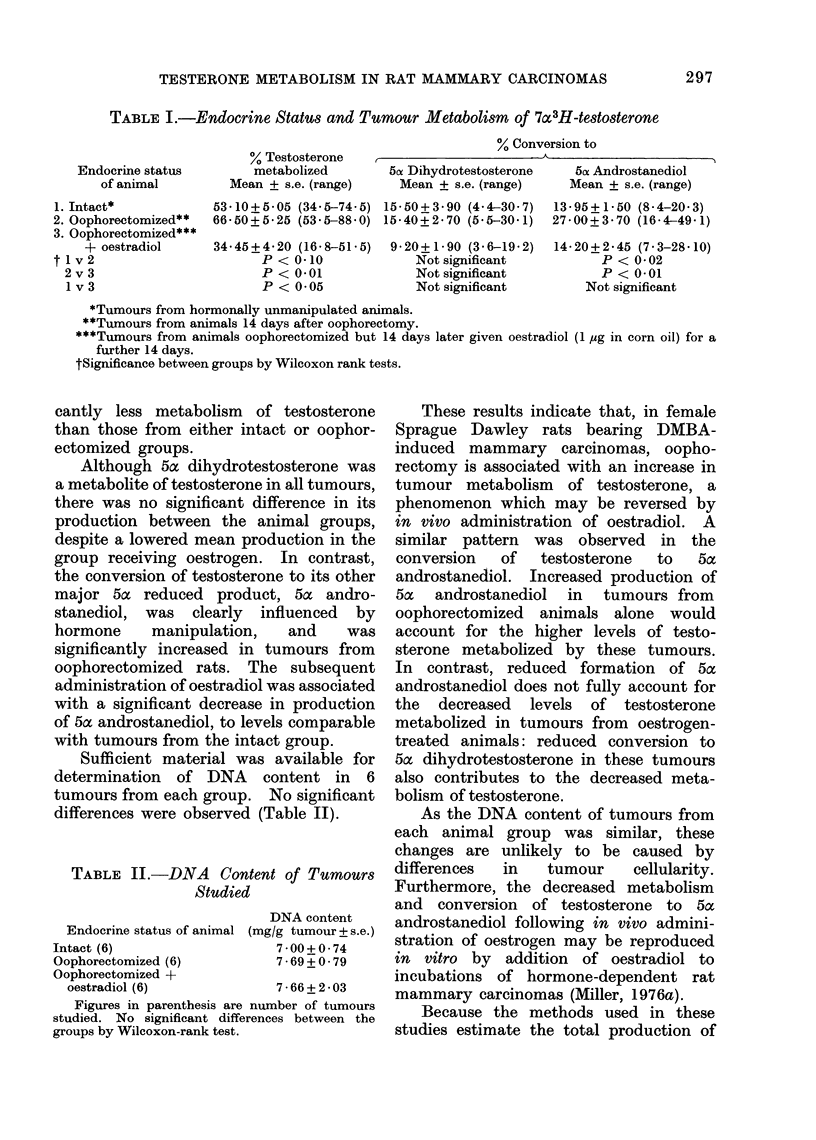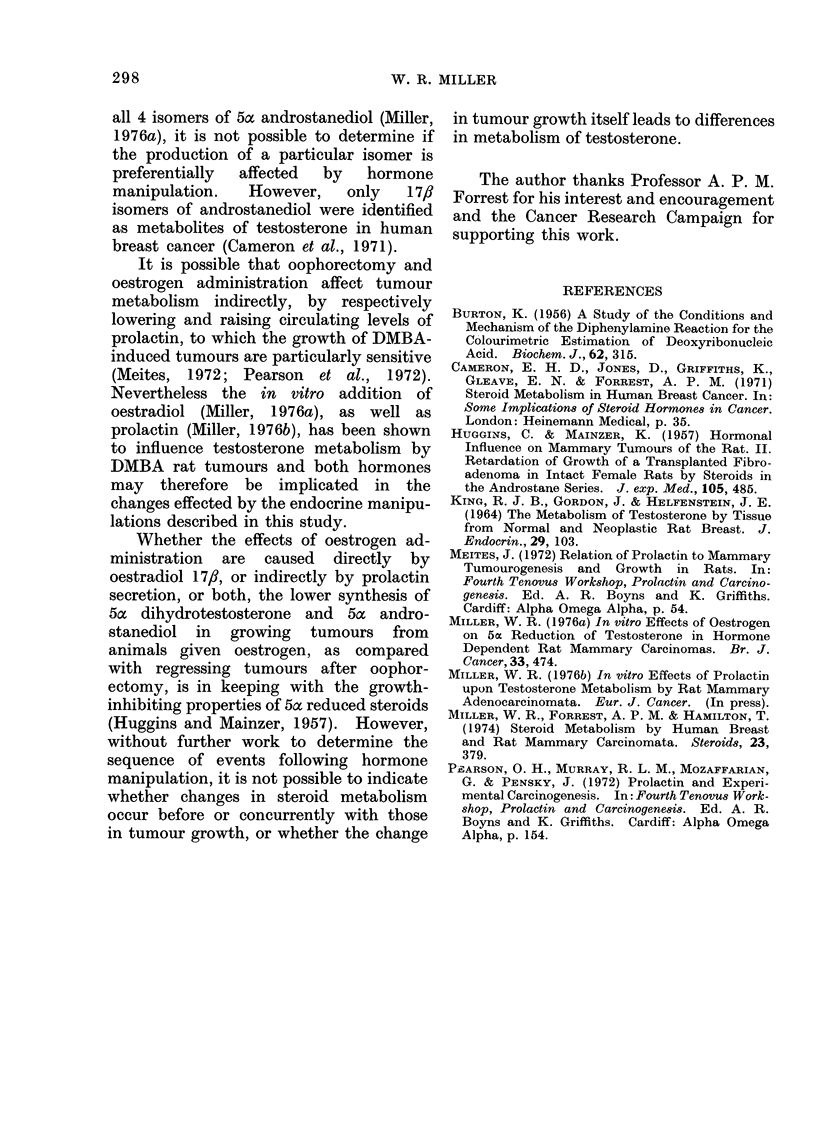# Hormonal status and testosterone metabolism of DMBA-induced rat mammary carcinomas.

**DOI:** 10.1038/bjc.1976.166

**Published:** 1976-09

**Authors:** W. R. Miller


*Br. J. Cancer* (1976) **34**, 296

***Short Communication***

### HORMONAL STATUS AND TESTOSTERONE METABOLISM OF DMBA-INDUCED RAT MAMMARY CARCINOMAS

W. R. MILLER

*From the Department of Clinical Surgery, University Medical School, Edinburgh EH8 9AG*

Received 12 March 1976 Accepted 25 May 1976

A MAJOR pathway of steroid metabolism in rat mammary carcinomas is the reduction of testosterone to 5α dihydrotestosterone and 5α androstanediol (King, Gordon and Helfenstein, 1964; Miller, Forrest and Hamilton, 1974). *In vitro* addition of oestradiol 17β to incubations of hormone-dependent rat mammary carcinomas is associated with an inhibition of 5α reduction of testosterone (Miller, 1976*a*). The aim of the present study was to determine if *in vivo* hormone manipulation was associated with similar changes.

A single i.v. injection of 5 mg 7-12-dimethylbenzanthracene (DMBA) was given to 24 randomly bred female Sprague Dawley rats at 50 days of age. The size of the tumours which were induced was monitored twice weekly by measuring with calipers 2 diameters at right angles. When the tumours were 2 × 2 cm in size, animals were allocated to one of 3 groups. Those in Group I were killed without further treatment, those in Group II were oophorectomized and killed 14 days later and those in Group III were oophorectomized but 14 days later received daily s.c. injections of 1 μg oestradiol in corn oil for a further 14 days, when they were killed. In all animals, oophorectomy led to regression of tumours; all those subsequently given oestradiol showed regrowth.

Tumours were harvested at death and treated at 0°C. Each was finely sliced in Krebs Ringer phosphate buffer pH 7·4 (10 ml/g tumour) and an NADPH-generating system (200 μmol glucose-6-phosphate, 30 μmol NADP and 50 u glucose-6-phosphate dehydrogenase/g tumour) and 7α $^3$H-testosterone (45 μCi/g tumour) added. The incubation systems were then shaken for 1 h at 37°C in an atmosphere of $O_2$. The reaction was stopped by adding methanol to 80% v/v and the incubations stored at −10°C until processed.

Before extraction, 500 μg of non-radioactive carrier testosterone (17β-hydroxy-4-androstene-3-one), 5α dihydrotestosterone (17β-hydroxy-5α-androstane-3-one) from Sigma Chemical Co. (St Louis, Mo.) and 5α androstanediol (5α-androstane 3β 17β diol) from Steraloids Inc. (N.Y.) were added to monitor recovery losses. The metabolites were extracted, separated into individual steroids and purified by thin layer chromatography as described previously (Miller *et al.*, 1974). The metabolism of testosterone by conversion to 5α dihydrotestosterone and 5α androstanediol were determined by measuring the percentage incorporation of radioactive label into the appropriate metabolites after correction for recovery losses.

The DNA content of the tumours was determined by a modification of the method of Burton (1956).

The pattern of testosterone metabolism by tumours from the 3 groups of animals is shown in Table I. There was an increased metabolism of testosterone in tumours harvested 14 days after oophorectomy, an effect reversed by administration of oestradiol 17β. Tumours from oestrogen-treated animals showed signifi-

TABLE I.—*Endocrine Status and Tumour Metabolism of 7α³H-testosterone*

| Endocrine status of animal | % Testosterone metabolized Mean ± s.e. (range) | % Conversion to: 5α Dihydrotestosterone Mean ± s.e. (range) | % Conversion to: 5α Androstanediol Mean ± s.e. (range) |
|---|---|---|---|
| 1. Intact* | 53·10±5·05 (34·5–74·5) | 15·50±3·90 (4·4–30·7) | 13·95±1·50 (8·4–20·3) |
| 2. Oophorectomized** | 66·50±5·25 (53·5–88·0) | 15·40±2·70 (5·5–30·1) | 27·00±3·70 (16·4–49·1) |
| 3. Oophorectomized*** + oestradiol | 34·45±4·20 (16·8–51·5) | 9·20±1·90 (3·6–19·2) | 14·20±2·45 (7·3–28·10) |
| † 1 v 2 | $P < 0{\cdot}10$ | Not significant | $P < 0{\cdot}02$ |
| 2 v 3 | $P < 0{\cdot}01$ | Not significant | $P < 0{\cdot}01$ |
| 1 v 3 | $P < 0{\cdot}05$ | Not significant | Not significant |

*Tumours from hormonally unmanipulated animals.
**Tumours from animals 14 days after oophorectomy.
***Tumours from animals oophorectomized but 14 days later given oestradiol (1 μg in corn oil) for a further 14 days.
†Significance between groups by Wilcoxon rank tests.

cantly less metabolism of testosterone than those from either intact or oophorectomized groups.

Although 5α dihydrotestosterone was a metabolite of testosterone in all tumours, there was no significant difference in its production between the animal groups, despite a lowered mean production in the group receiving oestrogen. In contrast, the conversion of testosterone to its other major 5α reduced product, 5α androstanediol, was clearly influenced by hormone manipulation, and was significantly increased in tumours from oophorectomized rats. The subsequent administration of oestradiol was associated with a significant decrease in production of 5α androstanediol, to levels comparable with tumours from the intact group.

Sufficient material was available for determination of DNA content in 6 tumours from each group. No significant differences were observed (Table II).

TABLE II.—*DNA Content of Tumours Studied*

| Endocrine status of animal | DNA content (mg/g tumour ± s.e.) |
|---|---|
| Intact (6) | 7·00±0·74 |
| Oophorectomized (6) | 7·69±0·79 |
| Oophorectomized + oestradiol (6) | 7·66±2·03 |

Figures in parenthesis are number of tumours studied. No significant differences between the groups by Wilcoxon-rank test.

These results indicate that, in female Sprague Dawley rats bearing DMBA-induced mammary carcinomas, oophorectomy is associated with an increase in tumour metabolism of testosterone, a phenomenon which may be reversed by *in vivo* administration of oestradiol. A similar pattern was observed in the conversion of testosterone to 5α androstanediol. Increased production of 5α androstanediol in tumours from oophorectomized animals alone would account for the higher levels of testosterone metabolized by these tumours. In contrast, reduced formation of 5α androstanediol does not fully account for the decreased levels of testosterone metabolized in tumours from oestrogen-treated animals: reduced conversion to 5α dihydrotestosterone in these tumours also contributes to the decreased metabolism of testosterone.

As the DNA content of tumours from each animal group was similar, these changes are unlikely to be caused by differences in tumour cellularity. Furthermore, the decreased metabolism and conversion of testosterone to 5α androstanediol following *in vivo* administration of oestrogen may be reproduced *in vitro* by addition of oestradiol to incubations of hormone-dependent rat mammary carcinomas (Miller, 1976*a*).

Because the methods used in these studies estimate the total production of all 4 isomers of $5\alpha$ androstanediol (Miller, 1976*a*), it is not possible to determine if the production of a particular isomer is preferentially affected by hormone manipulation. However, only $17\beta$ isomers of androstanediol were identified as metabolites of testosterone in human breast cancer (Cameron *et al.*, 1971).

It is possible that oophorectomy and oestrogen administration affect tumour metabolism indirectly, by respectively lowering and raising circulating levels of prolactin, to which the growth of DMBA-induced tumours are particularly sensitive (Meites, 1972; Pearson *et al.*, 1972). Nevertheless the *in vitro* addition of oestradiol (Miller, 1976*a*), as well as prolactin (Miller, 1976*b*), has been shown to influence testosterone metabolism by DMBA rat tumours and both hormones may therefore be implicated in the changes effected by the endocrine manipulations described in this study.

Whether the effects of oestrogen administration are caused directly by oestradiol $17\beta$, or indirectly by prolactin secretion, or both, the lower synthesis of $5\alpha$ dihydrotestosterone and $5\alpha$ androstanediol in growing tumours from animals given oestrogen, as compared with regressing tumours after oophorectomy, is in keeping with the growth-inhibiting properties of $5\alpha$ reduced steroids (Huggins and Mainzer, 1957). However, without further work to determine the sequence of events following hormone manipulation, it is not possible to indicate whether changes in steroid metabolism occur before or concurrently with those in tumour growth, or whether the change in tumour growth itself leads to differences in metabolism of testosterone.

The author thanks Professor A. P. M. Forrest for his interest and encouragement and the Cancer Research Campaign for supporting this work.

### REFERENCES

BURTON, K. (1956) A Study of the Conditions and Mechanism of the Diphenylamine Reaction for the Colourimetric Estimation of Deoxyribonucleic Acid. *Biochem. J.*, **62**, 315.

CAMERON, E. H. D., JONES, D., GRIFFITHS, K., GLEAVE, E. N. & FORREST, A. P. M. (1971) Steroid Metabolism in Human Breast Cancer. In: *Some Implications of Steroid Hormones in Cancer.* London: Heinemann Medical, p. 35.

HUGGINS, C. & MAINZER, K. (1957) Hormonal Influence on Mammary Tumours of the Rat. II. Retardation of Growth of a Transplanted Fibroadenoma in Intact Female Rats by Steroids in the Androstane Series. *J. exp. Med.*, **105**, 485.

KING, R. J. B., GORDON, J. & HELFENSTEIN, J. E. (1964) The Metabolism of Testosterone by Tissue from Normal and Neoplastic Rat Breast. *J. Endocrin.*, **29**, 103.

MEITES, J. (1972) Relation of Prolactin to Mammary Tumourogenesis and Growth in Rats. In: *Fourth Tenovus Workshop, Prolactin and Carcinogenesis.* Ed. A. R. Boyns and K. Griffiths. Cardiff: Alpha Omega Alpha, p. 54.

MILLER, W. R. (1976*a*) *In vitro* Effects of Oestrogen on $5\alpha$ Reduction of Testosterone in Hormone Dependent Rat Mammary Carcinomas. *Br. J. Cancer*, **33**, 474.

MILLER, W. R. (1976*b*) *In vitro* Effects of Prolactin upon Testosterone Metabolism by Rat Mammary Adenocarcinomata. *Eur. J. Cancer.* (In press).

MILLER, W. R., FORREST, A. P. M. & HAMILTON, T. (1974) Steroid Metabolism by Human Breast and Rat Mammary Carcinomata. *Steroids*, **23**, 379.

PEARSON, O. H., MURRAY, R. L. M., MOZAFFARIAN, G. & PENSKY, J. (1972) Prolactin and Experimental Carcinogenesis. In: *Fourth Tenovus Workshop, Prolactin and Carcinogenesis.* Ed. A. R. Boyns and K. Griffiths. Cardiff: Alpha Omega Alpha, p. 154.